# Investigating the influence of astringent compounds on oral lubrication and the protective role of proline-rich proteins

**DOI:** 10.1038/s41598-025-29493-2

**Published:** 2025-12-14

**Authors:** Ianis Ammam, Lucas Ouillon, Cyril Pailler-Mattéi, Roberto Vargiolu, Mélanie Lelièvre, Noémie Hermann, Nicolas Poirier, Fabrice Neiers, Francis Canon, Hassan Zahouani

**Affiliations:** 1 Ecole Centrale de Lyon, CNRS, ENTPE, LTDS, Ecully, UMR5513 France; 2 Université Bourgogne Europe, CNRS, Laboratoire Interdisciplinaire Carnot de Bourgogne ICB UMR 6303, Dijon, 21000 France; 3 Laboratoire de Tribologie et Dynamique des Systèmes, Ecole Centrale de Lyon, Université de Lyon, UMR-CNRS 5513, 36 Av. Guy de Collongue, Ecully, 69134 France

**Keywords:** Tissues, Mechanical engineering

## Abstract

Astringency, characterized by dryness and roughness in the mouth, is a major challenge for the acceptance of plant-based protein-rich foods. The mechanisms behind this sensation, particularly the role of mucins and the oral epithelium, remain unclear. This study investigates the impact of tannins, specifically epigallocatechin gallate (EgCG), on oral lubrication and examines the role of MUC1 protein in the lubrification and potentially in the astringency perception. The protective effect of proline-rich proteins (PRPs) is also explored. In vitro tribological tests were performed on four oral epithelial models expressing different MUC1 isoforms, using a reconstructed mucosal pellicle. A homemade biotribometer measured friction and dissipated energy to assess tannin-mucin interactions. Results confirm that EgCG disrupts epithelial lubrication, increasing frictional forces. However, MUC1 expression, particularly its structure, reduces these effects by preventing tannin aggregation and preserving lubrication. PRPs also enhance lubrication by binding tannins, limiting their interaction with mucins. This study highlights the roles of MUC1 and PRPs in oral lubrication in the presence of tannins.

**Supplementary Information:**

The online version contains supplementary material available at 10.1038/s41598-025-29493-2.

## Introduction

“Astringency is defined as the sensation of dryness and puckering in the mouth, particularly between the tongue and palate after swallowing”^1^. It is a complex sensation that is studied due to its relevance to many plant-based food products including wine, chocolate, tea, fruit and soya-based foods^2–5^. Moreover, the current global ecological context is motivating professionals in the agri-food industry to enhance the acceptance of sustainable plant-based food products, in view to increasing the incorporation of plant-based proteins into food. However, one of the main limitations of their acceptance is their green aroma, bitterness and astringency^6^.

Astringency is complex, and its definition and origin vary according to scientific hypotheses^7^. However, the components responsible for astringency are known: polyphenols^8^. The mechanisms of astringency follow two distinct modalities. The first seeks to elucidate astringency as a receptor-based sensation^5^, as astringent compounds can activate the trigeminal and gustatory nerves, giving rise to this very particular sensation^8,9^. Furthermore, a study by Simon, Hall and Schiffman^10^ showed that certain astringent compounds could inhibit the intracellular transport of ions in canine lingual epithelia, a mechanism which could contribute to the sensation of astringency in humans. However Green et al. showed that the sensation of astringency can be perceived on oral surfaces without a receptor, indicating that astringency is not only receptor-based^11^.

The second modality is a tactile phenomenon. In this hypothesis, the polyphenols bind, aggregate and precipitate specific salivary proteins, resulting in a modification of the lubrication on the surface of the oral mucosa^12,13^, i.e., a loss of lubrication, which leads to an increase in friction forces that activate the mechanoreceptors^4,5,8,14–16^. This loss of lubrication occurs when the complexes between salivary proteins and tannins aggregate on the surface of the oral mucosa on the mucosal pellicle (MP)^17^. The MP consists of a thin layer at the surface of the oral mucosa consisting of salivary proteins such as MUC5B, MUC7, and proline-rich proteins (PRP)^18,19^. These proteins are attached to the oral epithelium through interaction with an epithelial protein, namely, MUC1^20,21^. This MP contributes to the lubrication of the oral mucosa, but its lubricating power diminishes after exposure to astringent substances^7,22,23^.Thus, the aggregation of astringent components with salivary proteins causes a rupture of the MP on the surface of the oral cavity. This loss of lubrication gives the sensation of astringency which can be characterized by an increase in friction forces. Several authors have studied this depletion in lubrication on synthetic surfaces^5,24–26^. Agorastos et al. observed that the addition of polyphenols such as tannins and gallic acid led to an increase in friction caused by the breakdown of the salivary film on polydimethylsiloxane (PDMS)^7^. Other studies have also investigated the direct effect of certain foods such as wine, tea and milk without isolating the polyphenol on synthetic materials, using tribology tests and force measurement to monitor lubrication changes^27^. However, synthetic materials do not take into account that the properties and structures of the salivary film adsorbed on surfaces such as PDMS differ from human MP^20^. Among the salivary proteins prolin rich proteins (PRPs) appear to play a major role in interacting with astringent compounds. PRPs have a strong affinity for tannins, allowing them to bind to the latter and form stable protein complexes. Studies on these tannin-PRP complexes have led to the assumption that PRPs participate in the mucosal defence mechanism against tannins^28^. Consequently PRPs participate in mucosal protection by preventing tannins from penetrating the mucosa^12,29,30^. The aggregation of PRPs and their affinity of PRPs for tannins were explained in-depth by Canon et al^12^..

The experimental approaches developed in this work are based on the new hypothesis proposed by Canon et al. It proposes to integrate the role of the MUC1 protein in the physical mechanisms leading to astringency perception^20^. MUC1 is a transmembrane mucin anchored to the surface of the oral epithelium. Previous studies have already shown that MUC1 is involved in the salivary proteins anchored in the mucosa and participates in the lubrication of the epithelial cell surface^21,31,32^. MUC1 facilitates the anchoring of the salivary proteins included in the MP (MUC5B, MUC7, etc.). This new hypothesis^20^ suggests that MUC1 is involved in the perception of astringency through structural modifications, including the cleavage of its two subunits that trigger the transmission of a signal in addition to creating a protective mechanism against external aggression like tannins. MUC1 is organized in different domains: a membrane anchor part, a cleavable part (namely the SEA domain), and a richly glycosylated VNTR domain. The VNTR domain forms a shield that is difficult for astringent compounds to penetrate, due to strong steric hindrance.

The primary objective of this study is to explore the effect of tannins on the lubrication and involvement of the MUC1 protein in the physical mechanisms underlying astringency. This entails assessing whether the presence of MUC1 has any discernible impact, particularly with regard to its structural attributes. Lastly, the research scrutinizes the protective roles of PRPs to ascertain whether the presence of PRP mitigates the impact of tannins on lubrication.

**Fig. 1:**
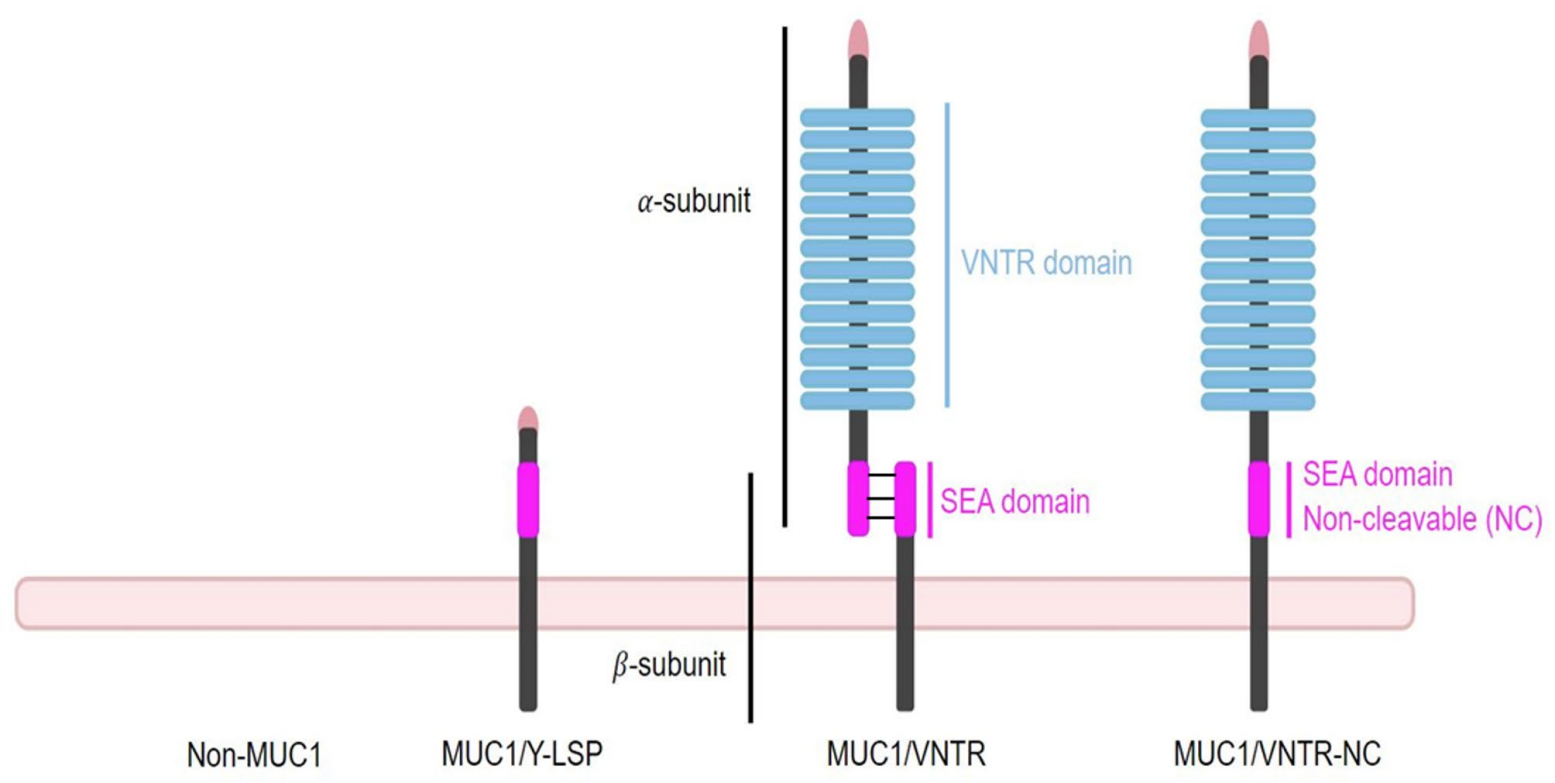
Schematic representation of the three MUC1 isoforms expressed in TR146-derived oral epithelium models.

In order to tackle these challenges, in vitro tribological experiments were performed on four oral epithelium models, aimed at simulating oral tribology such as interactions between proteins and tannins. The oral epithelium models expressing MUC1, as previously described^21,32^ consist of the TR146 cell line expressing different isoforms of MUC1 or not, along with a reconstituted MP after the incubation of TR146 cells with saliva. These isoforms, presented in the order shown in Fig. 1, include: an isoform with a shortened version of MUC1 lacking the VNTR domain; a wild-type-like isoform containing the VNTR region, a complete SEA domain, and two subunits (α and β); and a third isoform with an organization similar to the wild-type, but carrying a non-cleavable version of the SEA domain. These experiments were conducted using a homemade biotribometer^31^, making it possible to explore the tribological aspects of these complex interactions between MP and astringent compounds. Tribological parameters such as the friction coefficient, energy dissipated by friction, and the damaged surface area are measured and used as indicators of the lubrication state.

In addition to the control cell line lacking MUC1 expression, the three MUC1 isoforms differ in their structural organization, particularly in the VNTR and SEA domains. From left to right: a truncated isoform lacking the VNTR domain; a wild-type-like isoform containing the VNTR region, a full-length SEA domain, and two subunits (α and β); and an isoform structurally similar to the wild-type, but expressing a non-cleavable SEA domain.

## Results and discussion

### The impact of adding EgCG - Epigallocatechin gallate on the epithelium model lubrication

**Fig. 2:**
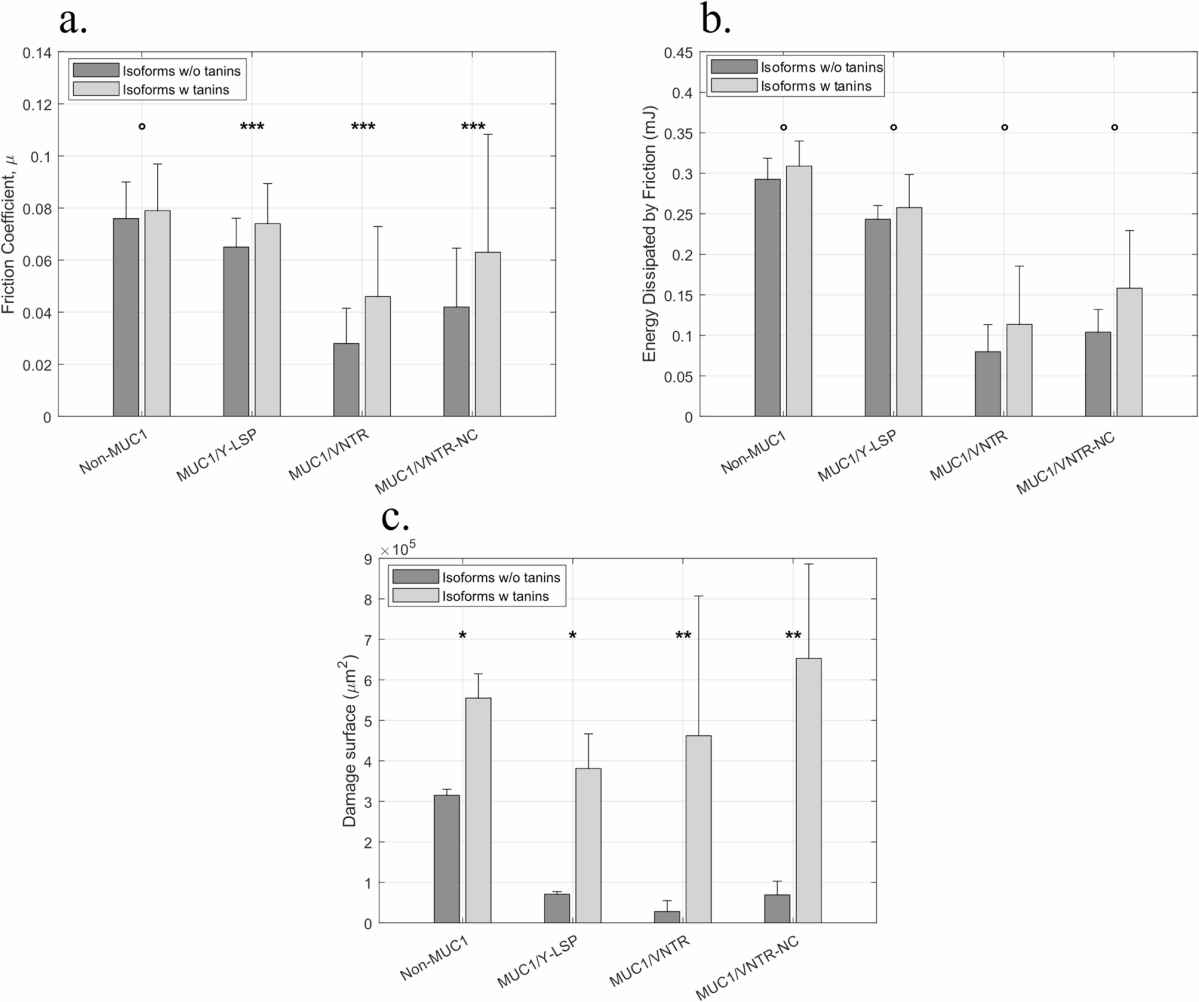
Impact of EgCG on the (**a**) Friction coefficient, (**b**) Energy Dissipated by friction, and (**c**) Damage surface for each MUC1 isoform – (Light grey for isoforms with tannins, Dark grey for isoforms without tannin) [mean values (*n* = 12) +/- standard deviation]. Statistical results were obtained using the method described in Sect. 3.5 - Signification codes: 0 < “***” < 0.001 < “**” < 0.01 < “*” < 0.05 < “.”.

These tribological tests provided insights into the impact of the astringent compound (here, EgCG) on the lubrication of the oral epithelium model. Figure 2 reveals that the addition of polyphenols leads to an increase in tribological parameters.

For the Non-MUC1 cell line the friction coefficient, the energy dissipated by friction and the damage surface were increased after the addition of EgCG (1mM). However, the friction coefficient increased only slightly from $\:0.076\:\pm\:0.014$ to $\:0.079\:\pm\:0.018$ $\:(p-value:\:^\circ\:)$, Fig. 2.a. For Ed, $\:0.29\pm\:0.03mJ$ to $\:0.31\pm\:0.03mJ$ (p-value: °), Fig. 2.b. The damage surface increased from $\:\left(3.15\pm\:0.15\right)\cdot\:{10}^{5}\mu\:{m}^{2}$ to $\:\left(5.55\pm\:0.60\right)\cdot\:{10}^{5}\mu\:{m}^{2}$ (p-value: *), Fig. 2.c.

Concerning the MUC1/Y-LSP isoform, the behaviour was the same, all the parameters were increased after the addition of EgCG. To illustrate this, the energy dissipated by friction increased from $\:0.24\pm\:0.02mJ$ to $\:0.26\pm\:0.04\:mJ$ (p-value: °), Fig. 2.b. Similarly, the other parameters, $\:\mu\:$ and the damage surface increased and both increases were statistically significant (p-value: *** and * resp.), Fig. 2.a.c.

With regard to the MUC1/VNTR isoform, the mechanical parameters increased after the inclusion of EgCG, for example, the damage surface which increased from $\:\left(0.28\pm\:0.27\right)\cdot\:{10}^{5}\mu\:{m}^{2}$ to $\:\left(4.62\pm\:3.45\right)\cdot\:{10}^{5}\mu\:{m}^{2}$ (p-value: **), Fig. 2.c. The friction coefficient and the energy dissipated by friction also increased (p-value: *** for $\:\mu\:$ and ° for Ed).

Finally, the friction forces ($\:\mu\:$ and Ed) and the damage increased in relation to the MUC1/VNTR-NC isoform. For instance, the friction coefficient rose from $\:0.042\pm\:0.023$ to $\:0.063\pm\:0.045$ (p-value: ***), Fig. 2.a. The other parameters also increased after the addition of EgCG, and these increases were statistically significant for damage (p-value: ° for $\:Ed$ and ** for damage).

**Fig. 3:**
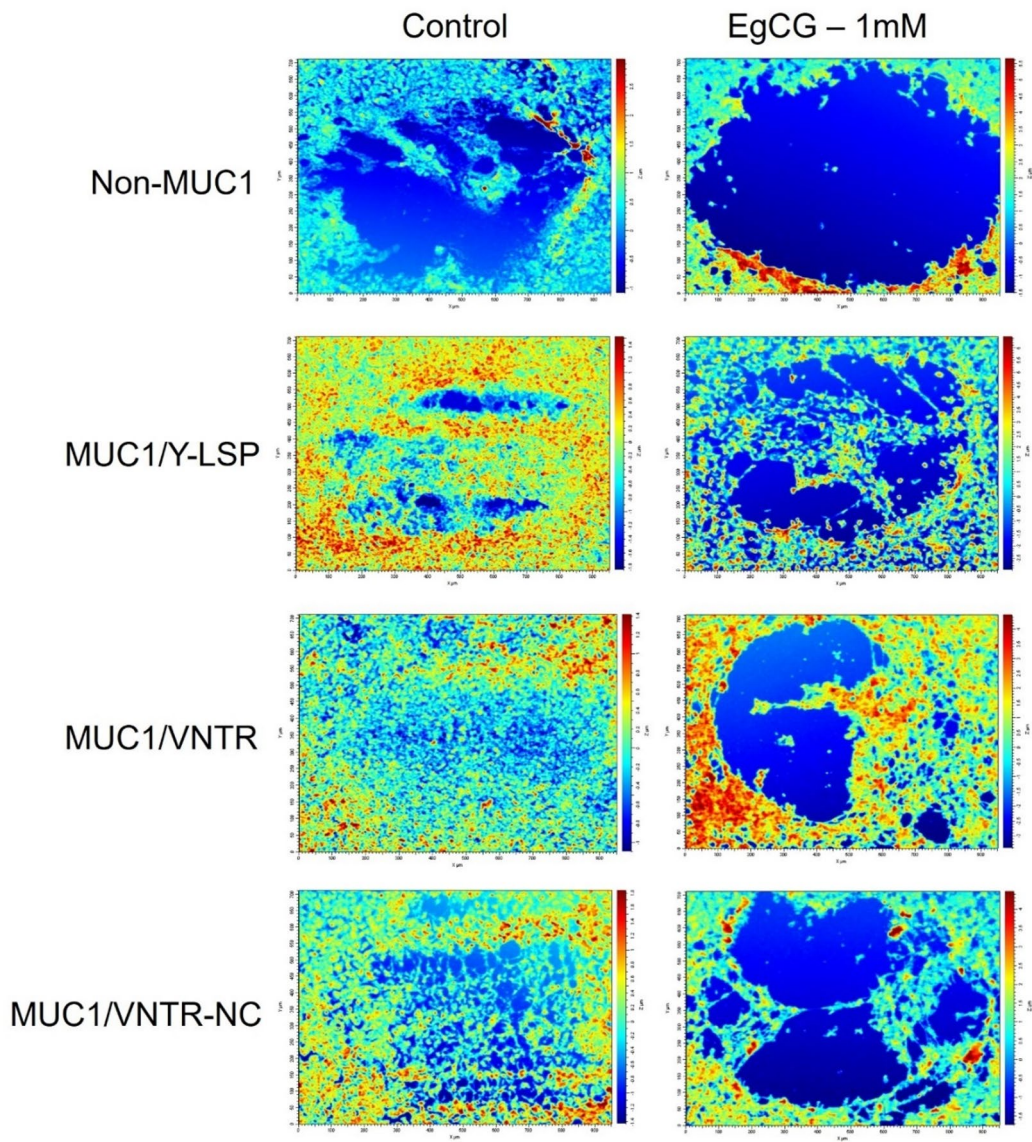
Interferometric Images (951 × 712 $\:{\upmu\:}{\text{m}}^{2}$) of the damage caused by friction on the epithelium models in two conditions: Control (without EgCG) and EgCG-1mM, for each MUC1 isoform.

Qualitatively, the images of the damage caused by friction show that the deteriorations are greater after the addition of EgCG, as can be seen in Fig. 3. The increase in damage serves as evidence of elevated friction forces during testing, consistent with a reduction in lubrication. The images are very clear, and this applies to each isoform. However, the level of damage is not the same depending on the isoform.

The experiments revealed that the addition of EgCG leads to an increase of the friction coefficient, energy dissipated and damage surface for four cell lines, indicating a decrease in the lubrication at the surface of the epithelium models. Previous research has demonstrated that the formation of the MP contributes to epithelium lubrication^21,31,32^. Our results show that the addition of tannins reduces the MP’s lubricating power. These results support a previous hypothesis suggesting that the addition of astringent compounds such as EgCG or gallic acid decreases the lubricating power of saliva on artificial surfaces^5,7,15^^24,25^^26^. This loss of lubrication had previously been mentioned by Bajec and Pickering^8^, who proposed that the aggregation of tannins on the oral surface can lead to an increase in friction forces.

However, all these studies were conducted on surfaces such as PDMS, which differs from in vivo oral surfaces in terms of protein-protein interaction. Our study shows the same results using oral epithelium models supporting this phenomenon in vitro, on the human oral epithelium. These models attempt to mimic the physiological surface and interface of the oral mucosa with the presence of the MUC1 protein and microplicae^21,32,33^, and also simulate the protein-protein interactions that contribute to in vivo oral lubrication^20^. Indeed, the new hypothesis has been proposed in the literature to explain the molecular mechanisms of astringency by incorporating the mucin MUC1 ^*20*^. This hypothesis suggests that astringent compounds form aggregates with MUC1, leading to the breakage of the two subunits at its SEA domain which leads to the disruption of the MP. This rupture gives rise to a mechanism potentially related to the perception of astringency. In general, the presence of aggregates results in a loss of lubrication.

The work presented here clearly and significantly demonstrates this increase in friction forces and damage caused by the addition of EgCG, and this applies to each cell line.

### The impact of adding EgCG – Exploring the role of the MUC1 structure

**Fig. 4:**
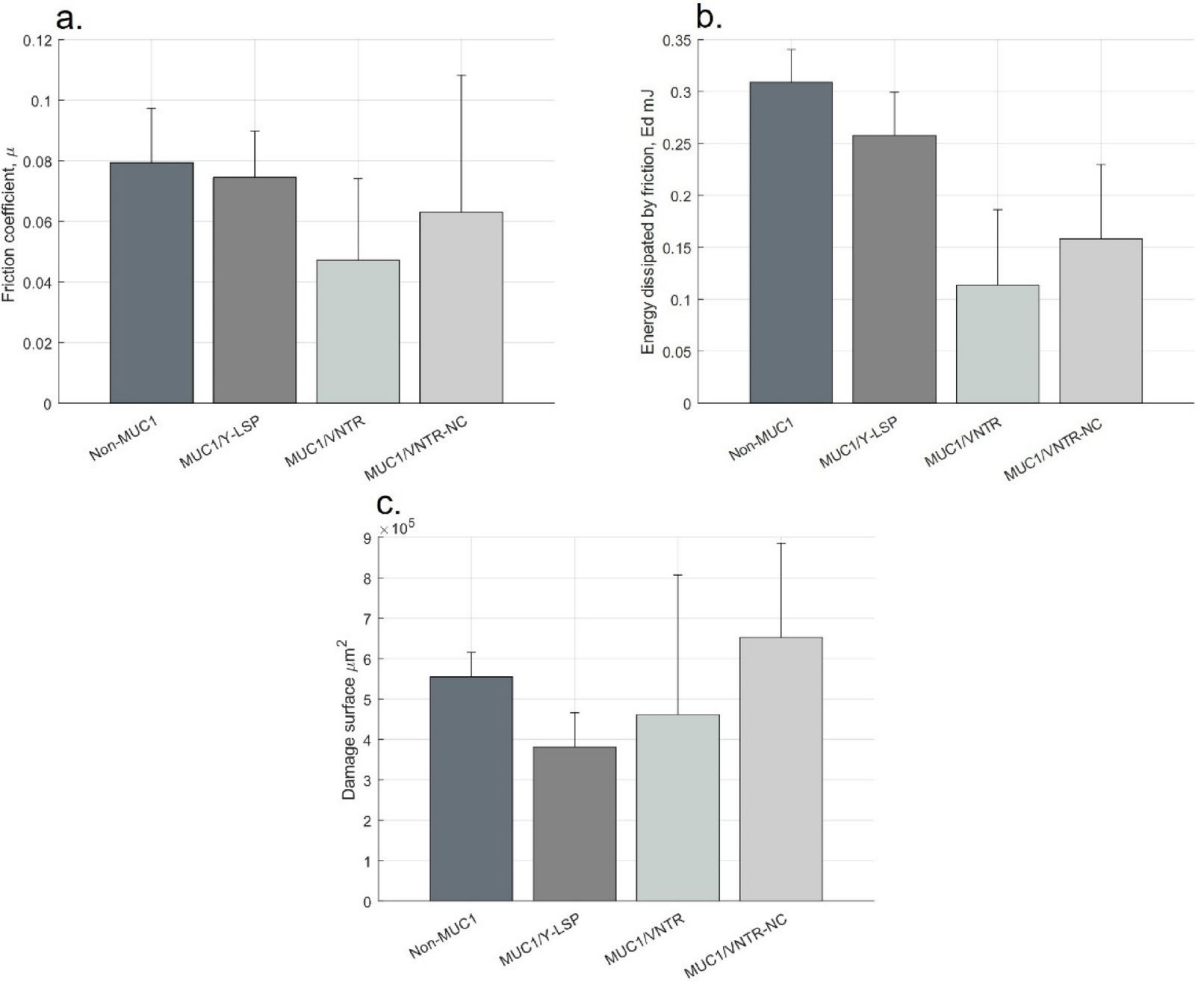
(**a**) Friction Coefficient, (**b**) Energy Dissipated by Friction and (**c**) Damage Surface of each isoform after exposure to EgCG [mean values (*n* = 12) +/- standard deviation]. Statistical results were obtained using the method described in Sect. 3.5 - Signification codes: 0 < “***” < 0.001 < “**” < 0.01 < “*” < 0.05 < “.”.

The comparison between isoforms after the addition of EgCG allows us to study the role of the MUC1 protein structure in the physical mechanisms underlying astringency, particularly the role of the cleavable SEA domain that contributes to epithelial protection^20,34,35^. The tests conducted in this work revealed that for the isoform possessing a cleavable SEA domain (MUC1/VNTR), friction forces are statistically lower than others (Non-MUC1, MUC1/Y-LSP and MUC1/VNTR-NC), Fig. 4.b.c. These findings indicate that the presence of a cleavable structure increases the protection of epithelial lubrication, probably subsequent to the cleaving of the SEA domain releasing the VNTR domain. Indeed, the dissociation of MUC1 into two subunits plays a role in the removal of the MP from the epithelium model surface, preventing the aggregation of tannin on the epithelium^34,36^. These mechanisms prevent the rupture of the mucosal pellicle and therefore result in a less significant increase in friction forces, Table 1.

Furthermore, our results revealed that the VNTR domain present in MUC1/VNTR and MUC1/VNTR-NC isoforms also seems to contribute to epithelial protection, preventing tannins from reaching the surface of the epithelium through the VNTR domain. Indeed, the friction coefficient and energy dissipated are lower with the presence of this VNTR domain (MUC1/VNTR and MUC1/VNTR-NC) compared to when it is absent (Non-MUC1, MUC1/Y-LSP), Fig. 4.

Finally, the truncated form of MUC1 also seems to have a slight influence. Given that we demonstrated in a previous study that MUC1 promotes lubrication^31^, it is likely that this phenomenon is also observed here. Despite the addition of tannins, the presence of MUC1 still promotes better lubrication compared to the Non-MUC1 isoform.

Regarding the damage caused by friction, the results are less conclusive, suggesting potential additional mechanisms possibly induced by the experimental protocol. Nonetheless, an increase in deterioration was still observed in the presence of tannins, indicating a loss of lubrication in general, see Fig. 4.

**Table 1:** Mann-Whitney test on the friction coefficient and dissipated energy, and Mann-Whitney test on damage surface among the 4 isoforms, to compare the effect of the MUC1 structure on interaction with EgCG. - Signification codes: 0 < “***” < 0.001 < “**” < 0.01 < “*” < 0.05 < “.”.

		Friction coefficient	Energy dissipated	Damage surface
Isoform	Isoform	$\:{p}_{value}$	$\:{p}_{value}$	$\:{p}_{value}$
Non-MUC1	MUC1/Y-LSP	°	**	***
Non-MUC1	MUC1/VNTR	***	***	°
Non-MUC1	MUC1/VNTR-NC	***	***	°
MUC1/Y-LSP	MUC1/VNTR	***	***	°
MUC1/Y-LSP	MUC1/VNTR-NC	***	***	**
MUC1/VNTR	MUC1/VNTR-NC	**	.	°

### The protective role of PRPs

**Fig. 5:**
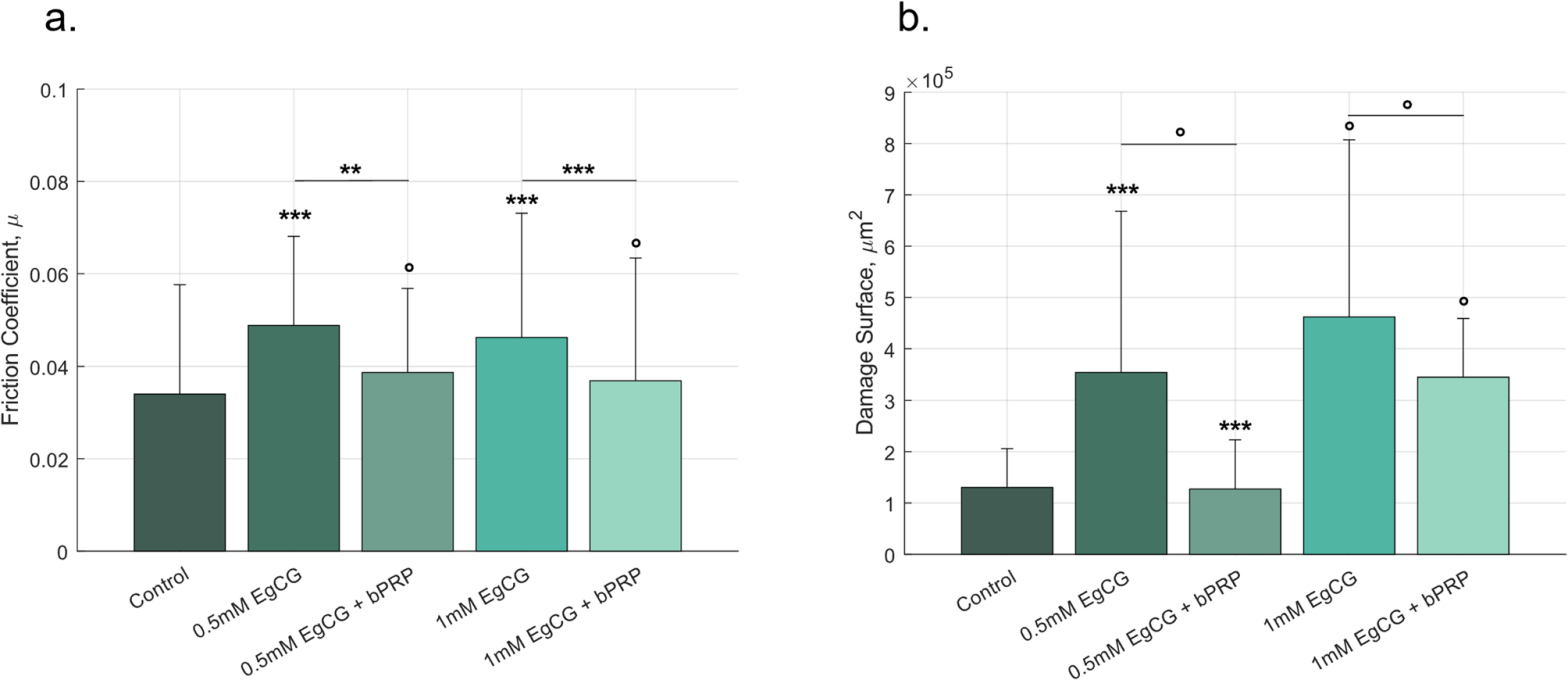
Impact of PRPs on a cell-based model. (**a**) Friction coefficient and (**b**) Damage surface after exposure of the cell-based model to EgCG (0.5mM and 1mM), with or without PRP (0.66mM). The significant code next to the error bar explains the difference with the control test (mean values (*n* = 12) +/- standard deviation). Statistical results were obtained using the method described in Sect. 3.5 - Signification codes: 0 < “***” < 0.001 < “**” < 0.01 < “*” < 0.05 < “.”.

This study marks an innovative exploration into the role of PRPs on the lubrication of the cell-based epithelium model. The friction coefficient and the damage to the epithelium models were reduced in the presence of PRP. This trend was observable for both concentrations of EgCG studied. For example, the friction coefficient fell from $\:0.046\:\pm\:0.027$ to $\:0.037\:\pm\:0.026$ for 1mM EgCG. In the same way, the damage surface decreased from $\:\left(4.60\pm\:3.45\:\right)\cdot\:{10}^{5}\mu\:{m}^{2}$ to $\:\left(3.45\pm\:1.14\:\right)\cdot\:{10}^{5}\mu\:{m}^{2}\:$also for EgCG 1mM. This reduction is statistically significant for the friction coefficient, but not significant for the damage surface, Fig. 5. The decrease is also observable for a lower concentration of EgCG (0.5mM).

Proline-rich proteins (PRPs) played a crucial role as a first line of defence against the toxicity of EgCG. The mucosa was preserved by preventing tannins from penetrating, thus reducing potential damage to the mucosal surface^12,29,30^. These protective mechanisms relied on the strong affinity of PRPs for tannins.

In this section, the protective aspect of PRPs was assessed through a tribological study. The addition of PRP before the introduction of EgCG allowed us to examine the protective effect of these proteins. The results demonstrated that the presence of PRPs significantly reduced the impact of tannins on lubrication for both concentrations (0.5 mM and 1 mM). For instance, the friction coefficient, reflecting the lubrication state of our epithelium model, was lower in the presence of PRP, indicating better lubrication of the MP compared to the absence of PRP. Indeed, PRPs protected the MP from the aggression of EgCG. Similarly, surface damage was less significant in the presence of PRP, confirming the protective role of this protein. These results are in line with the findings of Ployon et al., who showed that the addition of PRP IB5 limited the formation of mucin aggregates induced by EgCG (Ployon, 2018).

## Materials and methods

### Saliva collection

The research was conducted in strict accordance with the principles outlined in the Declaration of Helsinki, and with the informed consent of the participants. The saliva samples were approved by the Research Ethics Committee (CPP Est I. Dijon, #14.06.03, ANSM #2014-A00071-46). Saliva samples were obtained from individuals in good health who gave their written informed consent. The participants refrained from drinking, smoking or eating for a period of 2 h before sample collection. The donors let the saliva flow naturally into plastic containers to obtain unstimulated saliva. To prevent the degradation of the biological material, the saliva collected was either used immediately or promptly frozen in liquid nitrogen.

### MUC1 isoforms

Tribological measurements were carried out on an in vitro model of the oral epithelium. These models were based on the TR146 cell line. Four different TR146 cell lines were used (see Table 2). Three of these cell lines were derived from a parental TR146 cell line, which does not express MUC1, and were transfected with three different genes coding for three MUC1 isoforms. These four in vitro models were described in more detail further in the following study^31^. A summary of the characteristics of these four cell lines is presented in Table 2.

**Table 2:** Description of the cell lines used in this work.

Isoforms	Properties	References
Non-MUC1 (TR146 Parental cell line)	Lacks expression of MUC1. Native form provided by ECACC.	European Collection of Cell Culture, Salisbury, Wiltshire, UK
MUC1/Y-LSP	Expresses an MUC1 isoform lacking the VNTR domain and having a truncated SEA domain.	^21^
MUC1/VNTR	Encodes an isoform of MUC1, closed to the wild isoforms, with 2 subunits $\:\alpha\:$ and $\:\beta\:$, the VNTR domain and a full SEA domain (cleavable).	^32^
MUC1/VNTR-NC	Similar to MUC1/VNTR but with only one subunit has it contains a truncated SEA domain, which preclude the cleavage of MUC1 into two subunits.	

### Cell culture

The cell culture routines used for cultivating the four cell lines were the same as in our previous work^31^. A medium referred to as basic was used to cultivate cells from the parental line: Non-MUC1. The medium is DMEM/F12 + GlutaMax (11559726, Gibco, TermoFischer, Germany) (1:1, v: v) supplemented with 10% foetal bovin serum (FBS, 10500064 et A5209402, Gibco, TermoFischer, Germany) ans 100 units/ml penicillin, 100 mg/ml streptomycin in T75 flasks (all from Gibco, TermoFischer, Germany). This medium was supplemented with 2,5 mg/mL of Geneticin (10131027, Gibco, TermoFischer, Germany) to cultivate the MUC1/Y-LSP cell line. Finally, the basic medium was supplemented with 2 µg/mL of Zeocin (ant-zn-05, Invivogen, France) to cultivate the cell line with a VNTR domain: MUC1/VNTR and MUC1/VNTR-NC. Cells were subcultured every 4 days and the medium was changed every 2 days. Cells were incubated at 37 °C in a humidified atmosphere containing 7.5% CO_2_.

Glass slides, previously coated with Poly-D-Lysine (Gibco, TermoFischer, Germany), were used as substrate for the epithelium model. The in vitro epithelium model comprised the cell layer that covered the substrate (glass slide). Mechanical tests were conducted on this model, varying with MUC1 isoforms.

### Mucosal pellicle reconstruction

The mucosal pellicle was reconstructed on the cells monolayer surface following a 2-hour incubation with saliva and growth medium (1:1). After incubation, the cells were rinsed twice with PBS to eliminate the saliva that had not adhered to the epithelium, in order to retain only the MP anchored to the mucosal model as suggest in a previous study^37^.

### Epigallocatechin gallate (EgCG) solution and exposure to the epithelium model

EgCG was purchased from Sigma Aldrich (USA). The EgCG concentration used for the friction test was 1 mM. This concentration was chosen to work above the sensory detection threshold of EgCG, measured at 0.54mM^38^.

Before the friction test and after MP formation, EgCG was added to the cell-based model. The EgCG solution (1mM EgCG in PBS) covered the epithelium model in the incubator for 10 min. After 10 min, the solution was removed and the friction test was performed.

### Exposure of the model to EgCG with PRP solution

The protective role of PRPs against EgCG aggression was evaluated. The PRP was produced, purified as described previously^39^.

After the formation of the MP, the epithelium model was covered with 1 mL of PRP solution containing 0.66 mM of PRP in PBS for 5 min^40^. Subsequently, EgCG was added to the cell-based model at three concentrations: 0 (corresponding to the control condition), 0.5, and 1 mM, also for 5 min, following the same procedure as described in Sect. 3.5. After that, the friction test was performed. This test was conducted exclusively on the MUC1/VNTR isoform.

### Tribological measurement

The tribological methodology employed in this study involves monitoring the changes in friction forces at the surface of the oral epithelium model under various conditions. All the tribological measurements were conducted using a homemade device^31^. This apparatus was utilized to assess the lubrication properties of the sample by calculating the friction coefficient and the energy dissipated by friction.

During the tests, a normal load of 0.5 $\:mN$ was applied to the oral epithelium model with an indenter, and this set point was maintained and controlled using a PID regulator throughout the test to avoid the viscoelastic effects of the materials used. The spherical silicone indenter had a diameter of 6 mm and a reduced Young’s modulus $\:{E}^{*}$ of ~ 150$\:kPa$ measured by indentation. The tribological test was performed once the set point was reached. The tangential linear speed was 300 $\:\mu\:m/s$, and each test consisted of 10 cycles. These values were chosen to avoid damaging the cell layer during the test and to ensure that the test was conducted on the cell layer and not on the substrate. During the tests, the normal and tangential forces were recorded to calculate the friction coefficient and the energy dissipated by friction^31^. These parameters were used as indicators of the lubricating state of the cell-based model. The device was specifically designed to mimic the physiological movement of oral tissues.

### Damage analysis

The damage caused by friction was assessed in this study. Considering that lubrication reduces friction forces, the damage served as an interesting indicator of the lubrication of the epithelium model.

Immediately after the friction test, each in vitro epithelium model was fixed with a 4% solution of Paraformaldehyde (PFA, Thermo Fisher Scientific, USA) in PBS (Thermo Fisher Scientific, USA) and dehydrated in four successive ethanol baths (70%, 80%, 90%, 100%). The friction trace was captured by optical interferometry (Bruker, USA).

The damage analysis method was used following the procedure described by Ammam et al^31^..

### Statistical analysis

A statistical analysis was performed to ensure statistically significant differences. Firstly, a Kruskall-Wallis test was performed to study the overall similarity, providing an initial indication of the overall similarity between the groups. Then, Mann-Whitney tests were conducted, comparing each pair of samples pairwise. The objective was to determine if statistically significant differences existed between these specific groups. Also, a Bonferroni correction was applied to minimize errors in p-values caused by the repetition of tests.

These tests provided us with the p-value, indicating the level of confidence we can place in the results, with a risk level of $\:\alpha\:\le\:0.05$. The data analysis was performed using Matlab (R2021b, École Centrale de Lyon Licence). Significance codes:

$$\:p\:=\:\:0\:<\:***\:<\:0.001\:<\:**\:<\:0.01\:<\:*\:<\:0.05\:<^\circ\:\:<\:0.1\:<\:.\:<\:1$$

## Conclusion

This study explored the molecular mechanisms of astringency and its effects on lubrication. Tribological tests on our epithelial models allowed us to assess their lubrication state. The advantage of using these models is that they replicate protein-protein or protein-tannin interactions observed in vivo, thereby facilitating the examination of MUC1’s specific function and structure in the physical processes underlying astringency perception. The significance of this study is that it presents biological hypotheses across scales, from the microscopic to the macroscopic, thus validating various hypotheses proposed in the existing literature.

These results revealed the detrimental impact of EgCG on epithelial lubrication, thereby confirming existing hypotheses in the literature. The addition of tannins leads to decreased lubrication, a loss quantified by our tribological parameters, which showed an increase in frictional forces and post-mortem damage.

The expression of MUC1 in our models allowed analysing its role in these molecular mechanisms. The specific structure of MUC1 mucin appears to mitigate the effect of EgCG, likely through the cleavage of the SEA domain, which releases the VNTR domain^20,34^. The dissociation of MUC1 into two subunits contributes to the removal of certain mucin proteins from the epithelial surface, preventing the aggregation of EgCG^34,41^. Furthermore, the VNTR domain of MUC1/VNTR and MUC1/VNTR-NC isoforms seems to contribute to protection by preventing tannins from reaching the epithelial surface and forming aggregates. These mechanisms prevent the breakdown of the mucosal layer and consequently reduce the harmful effects of EgCG compared to other isoforms.

In addition to the protective mechanisms of MUC1, our methodology identified the active role of PRPs in protecting epithelia from tannins. PRPs bind to tannins before they can aggregate with salivary mucin proteins, thereby preserving lubrication. Our tests demonstrated a reduction in tribological parameters in the presence of PRPs, indicating improved lubrication. This opens avenues for studying other proteins involved in epithelial lubrication.

## Supplementary Information

Below is the link to the electronic supplementary material.

Supplementary Material 1

Supplementary Material 2

Supplementary Material 3

Supplementary Material 4

Supplementary Material 5

Supplementary Material 6

Supplementary Material 7

Supplementary Material 8

Supplementary Material 9

Supplementary Material 10

Supplementary Material 11

Supplementary Material 12

Supplementary Material 13

Supplementary Material 14

Supplementary Material 15

Supplementary Material 16

Supplementary Material 17

Supplementary Material 18

Supplementary Material 19

Supplementary Material 20

Supplementary Material 21

Supplementary Material 22

Supplementary Material 23

Supplementary Material 24

Supplementary Material 25

Supplementary Material 26

Supplementary Material 27

Supplementary Material 28

Supplementary Material 29

Supplementary Material 30

Supplementary Material 31

Supplementary Material 32

Supplementary Material 33

Supplementary Material 34

Supplementary Material 35

Supplementary Material 36

Supplementary Material 37

Supplementary Material 38

Supplementary Material 39

Supplementary Material 40

Supplementary Material 41

Supplementary Material 42

Supplementary Material 43

Supplementary Material 44

Supplementary Material 45

Supplementary Material 46

Supplementary Material 47

Supplementary Material 48

Supplementary Material 49

Supplementary Material 50

Supplementary Material 51

Supplementary Material 52

Supplementary Material 53

Supplementary Material 54

Supplementary Material 55

Supplementary Material 56

Supplementary Material 57

Supplementary Material 58

Supplementary Material 59

Supplementary Material 60

Supplementary Material 61

Supplementary Material 62

Supplementary Material 63

Supplementary Material 64

Supplementary Material 65

Supplementary Material 66

Supplementary Material 67

Supplementary Material 68

Supplementary Material 69

Supplementary Material 70

Supplementary Material 71

Supplementary Material 72

Supplementary Material 73

## Data Availability

All data generated or analyzed during this study are included in this published article and its supplementary information files.
